# A Possible Role of Amyloidogenic Blood Clotting in the Evolving Haemodynamics of Female Migraine-With-Aura: Results From a Pilot Study

**DOI:** 10.3389/fneur.2019.01262

**Published:** 2019-11-26

**Authors:** Sulette de Villiers, Janette Bester, Douglas B. Kell, Etheresia Pretorius

**Affiliations:** ^1^Department of Physiology, Faculty of Health Sciences, University of Pretoria, Pretoria, South Africa; ^2^Department of Biochemistry, Institute of Integrative Biology, University of Liverpool, Liverpool, United Kingdom; ^3^Department of Physiological Sciences, Faculty of Science, Stellenbosch University, Stellenbosch, South Africa

**Keywords:** migraine-with-aura, coagulation, fibrin(ogen), β-amyloid, thromboelastography, eryptosis

## Abstract

**Introduction:** Migraine is a debilitating primary headache disorder with a poorly understood aetiology. An extensive body of literature supports the theory of migraine as a systemic vascular inflammatory disorder characterised by endothelial dysfunction. It is also well-known that chronic inflammation results in an excessive burden of oxidative stress and therefore cellular dysfunction. In this study the effects of excessive oxidative stress through the phases of female migraine-with-aura (FMA) were evaluated by examining the health of the systems of haemostasis.

**Methods:** Blood was obtained from 11 FMA patients at baseline and during the headache phase of migraine, as well as from 8 healthy age-matched female controls. Samples were analysed using thromboelastography (TEG) to evaluate viscoelastic profiles, light microscopy for erythrocyte morphology, Scanning Electron Microscopy (SEM) for erythrocyte and fibrin clot structure, confocal microscopy for β-amyloid detection in fibrin clots.

**Results:** Viscoelastic profiles from platelet poor plasma showed decreased clot reaction times in FMA at baseline (95% CI [5.56, 8.41]) vs. control (95% CI [7.22, 11.68]); as well as decreased time to maximum thrombus generation for the same comparison (95% CI [6.78, 10.20] vs. [8.90, 12.96]). Morphological analysis of erythrocytes indicated widespread macrocytosis, poikilocytosis and eryptosis in the migraineurs. Analysis of fibrin networks indicated that this hypercoagulability may be a result of aberrant fibrin polymerisation kinetics caused by the adoption of a β-amyloid conformation of fibrin(ogen).

**Conclusion:** The results reaffirm the hypercoagulable state in migraine, and would suggest that this state is most likely a result of a systemic inflammatory state which induces oxidative damage to both erythrocytes and fibrin(ogen) in female episodic migraine-with-aura. Furthermore, if the amylodogenic changes to fibrin(ogen) were observed in a larger cohort, this would support theories of micro-embolisation in migraine-with-aura.

## Introduction

Migraine with aura is a debilitating primary headache disorder diagnosed by specific criteria determined by the International Headache Society (1). Of greatest significance are the unilateral pulsatile pain sensation and the transient neurological disturbances affecting vision, sensation, speech, language comprehension, or motor skills (1).

The pulsating nature of migraine headache implicates cerebral vasculature in pathogenesis, and indeed it is known that altered cortical blood flow in migraine with aura results in the phenomenon known as Cortical Spreading Depression (CSD) occurring spontaneously before the onset of headache (1–5). CSD results from a release of the neurotransmitter glutamate in a domino effect, which results in a wave of glutamatergic neuron excitation followed by a wave of inhibition. This results in the vasodilation of the meningeal vasculature with release of inflammatory neuropeptides, such as calcitonin gene-related peptide, substance P, and neurokinin A (3, 6). This inflammatory process promotes vasodilation of the cerebral arteries and dural venous sinuses, as well as inducing inflammatory cytokine production and activation of mast cells and leukocytes. The end result is the sensitisation of the peripheral nociceptors of the trigeminovascular system and the headache phase of migraine, characterised by pulsating pain that is aggravated by any activity that increases intracranial pressure (7–11).

A model of endothelial dysfunction in the trigeminovascular system has emerged in migraine research. Endothelial dysfunction induces an inflammatory response which results in oxidative stress and a hypercoagulable state marked by increased platelet reactivity, altered erythrocyte morphology and metabolism, and increased fibrinogen levels (5–10, 12–14). In this model it is inherently suggestive that haemostasis would be affected through the phases of migraine.

Red blood cells can act as free radical scavengers preventing oxidative damage of body tissues and are unique cellular indicators of the presence of an inflammatory pathology as well as the body's response to such an event (15–17). Prolonged exposure to oxidative elements results in a greater amount of free radicals being produced than can be neutralised, leading to oxidative damage of red blood cells and especially of the phospholipid bilayer membranes. In pathological states and under oxidative stress the normally asymmetrical distribution of lipids between inner and outer sides of the bilayer is disturbed, resulting in membrane scrambling (16, 18, 19).

The role of fibrin(ogen) in migraine pathophysiology has not been well-explored (20). Fibrinogen is exceptionally sensitive to oxidation, serving as a free radical scavenger and protecting other plasma proteins from damage (21–23). Although vital, this function results in altered fibrin structure in individuals suffering from chronic systemic inflammation who are burdened by greater degrees of oxidative stress. A variety of mechanisms by which this structural alteration may occur have been proposed, while the ultimate result is agreed to be densely matted networks (21, 22, 24).

We thus considered it worthwhile to investigate the ultrastructure of the fibrin networks of FMA patients, and to investigate whether the alteration to fibrin networks seen under oxidative stress might be the result of an amyloidogenic process active in the pathophysiology of migraine with aura.

Furthermore, we considered the changes that altered cortical blood flow and recurrent inflammatory dysregulation could have on rheology. As such we evaluated erythrocyte structure and platelet activation as vital indicators of overall health in the FMA cohort (16).

## Materials and Methods

### Study Design

This was a case control observational pilot study. The primary objective was to determine if the blood of female episodic migraine-with-aura indicated systemic inflammation and a state of changing coagulability through the phases of migraine. Secondary objectives were to evaluate fibrin networks under scanning electron microscopy for evidence of altered polymerisation and clotting dynamics; to critically evaluate erythrocyte morphology for indicators of oxidative stress using light and scanning electron microscopy of native whole blood; to evaluate viscoelasticity of whole blood and platelet poor plasma through the phases of migraine using thromboelastography; and to determine the presence of β-amyloidogenic alterations to fibrin fibrils by using confocal microscopy.

Although power calculations were made by a biostatistician of the Research Office of the Faculty of Health Sciences, University of Pretoria, sample size was ultimately limited by recruitment time. Statistical analyses were carried out at the advice of the biostatistician as two-tailed *t*-tests and Kruskal-Wallis ANOVA with Dunn's post-tests to correct for multiple comparisons. Red blood cell morphology parameters were additionally analysed using linear mixed modelling and quantile regression, respectively, to consider for between-patient variation, using STATA15. Parameters not conforming to a Gaussian distribution were analysed by Wilcoxon matched-pairs signed rank tests. Owing to the nature of the measurement and a current lack of standards for red blood cell axial ratios, as well as the need to correct for multiple testing, it was decided to test the axial ratios at a significance level of 95% (*P* < 0.05) and then divide by three for a final *P*-value of 0.0167. Unless stated otherwise analyses were performed using GraphPad Prism 6, with minimal significance defined as *P* < 0.05 (CI = 95%) (25–27). Normality was evaluated using D'Agostino and Pearson tests. Confounding factors were very strictly excluded from the study in order to avoid haemostatic or rheological changes that could not be ascribed to episodic migraine-with-aura or the treatment thereof.

Patients were recruited under informed consent from a private migraine clinic as well from the staff of the Faculty of Health Sciences at the University of Pretoria. Inclusion in the study was dependent on diagnosis as an episodic migraine sufferer as detailed by the International Headache Classification III guidelines and under the guidance of a neurologist specialised in migraine treatment (1). The following exclusion criteria directed recruitment: age younger than 18 years; smoking; use of chronic medication other than that which has been prescribed for the treatment of episodic migraine; prior history of cardiovascular disease, transient ischaemic attack, asthma or auto-immune disease. Stage of ovarian and menstrual cycles, and method of contraception were not controlled for in this pilot. Blood samples were obtained from the participants “at baseline” (defined here as the interictal period) and during the headache phase of a migraine attack.

### Viscoelasticity

Thromboelastography was performed on whole blood and on platelet poor plasma (obtained by centrifugation of citrated native blood and thawed after storage at −80°C) at baseline and during the headache phase of migraine using a TEG 5000 computer-controlled device (Haemonetics Corp., Niles, IL, USA) and following manufacturer's instructions. This method was used to monitor the viscoelastic profiles of migraineurs at baseline and during the headache phase of migraine.

### Erythrocyte Morphology

Whole blood collected in citrate tubes was analysed under light and scanning electron microscopy. Methylene blue/eosin (MB/E) differentially stained peripheral blood smears were viewed under a 100 X oil-immersion objective of a Zeiss AXIO Imager M2. Cellanyzer v 1.1 software was used to determine axial ratios and circumferences of red blood cells. SEM samples were viewed using a Zeiss Cryo CR-Beam Ultra-resolution SEM at 1 kV.

### Fibrin Morphology and Amyloidogenesis

Fibrin(ogen) structure and polymerisation kinetics were investigated using scanning electron microscopy and fluorescent microscopy for amyloid mutation. Fibrin clots were prepared for SEM by the addition of reconstituted human thrombin to platelet poor plasma (PPP). Clots were viewed under 35 k magnification using a Zeiss Cryo CR-Beam Ultra-resolution SEM at 1 kV magnification and were analysed for 8-bit pixel intensity using the histogram function of Image J v2.0 FIJI (National Institutes of Health, USA). Amyloid fibrils show an unbranched ribbon morphology consisting of laminated β-sheets running perpendicular to the long axis. It is this structure that allows for the binding of the benzothiazole dye Thioflavin T(ThT), a potent *in vitro* fluorescent marker when bound to amyloid fibrils. Where detection of amyloid by fluorescent microscopy is concerned ThT has shown superiority over congo red and methyl violet, showing increased sensitivity and efficiency, with enhanced excitation maximum (450 nm) and wide emission spectrum of 508 to 570 nm (28). Fibrinogen was treated by addition of thioflavin T to a final concentration of 0.025 mmol/μl (Abcam, ab120751). Fibrin clots were prepared by addition of thrombin to the treated PPP. Clots were viewed under 63 X oil-immersion objective of a Zeiss LSM 880 with Airyscan using the 488 nm laser with a 458 nm excitation filter to overcome a reflectance issue.

## Results

A total of thirteen female migraineurs with aura episodic migraineurs were recruited for participation in this study. Of these individuals, one was excluded owing to the later diagnosis of a medical condition that met exclusion criteria, while another was lost to follow-up. Mean age of the remaining eleven was 37.4 ± 12.1 years, with average age of migraine with aura onset of 18.6 ± 7.6 years at an average frequency of 2.9 ± 1.5 migraines per month. The participants subjectively rated their pain levels on a scale of 1 (lowest intensity) to 10 (highest intensity). Using this system the participants scored mild migraines an average of three, while severe headaches were scored an average of nine. Regarding aura symptomatology: 90.9% reported visual disturbances, 81.8% reported sensory disturbances, 54.5% reported motor disturbances, 36.4% reported difficulty speaking, and 18.2% reported difficulty understanding speech. Mean age of the female controls was 39.9 ± 11.4 years.

### Viscoelasticity

**Figure 1:**
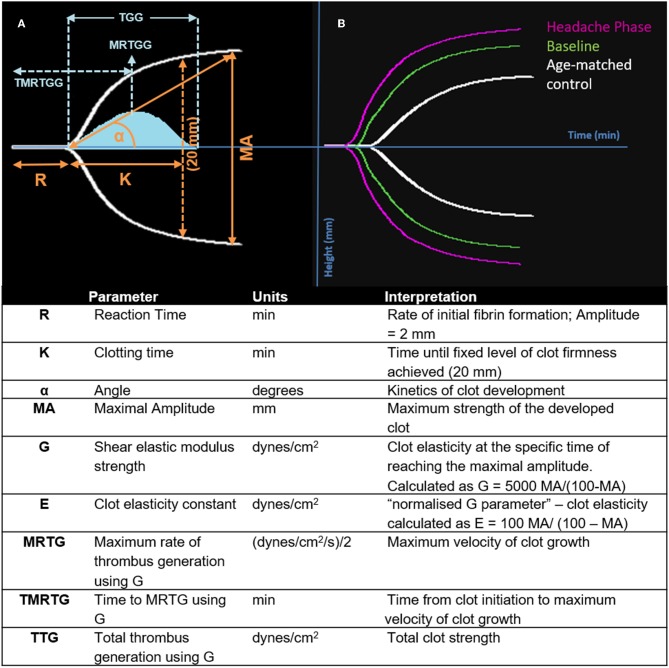
Interpretation of the TEG® tracing (blue in **A**), velocity curve (blue in **B**), and parameters produced for FMA at baseline and headache phase compared to age-matched healthy controls. The velocity curve parameters (MRTG, TMRTG, and TTG) are calculated using the G value of the TEG® tracing.

**Figure 2:**
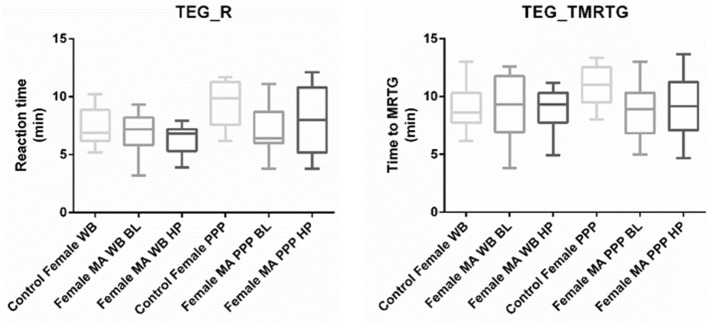
Tukey box plots for clot reaction time (R, min) and time to maximum thrombus generation (TMRTG, min). Both parameters were significantly decreased in PPP samples obtained from migraine at baseline compared to PPP from healthy age-matched controls.

Nine TEG® parameters were measured to determine the viscoelastic profiles of healthy controls and FMA patients. The tracings produced, their interpretation, and the nine parameters of interest are shown in Figure 1 (29, 30). No significant differences were found in whole blood samples drawn during the headache phase or baseline compared to healthy age-matched controls. Analysis of PPP samples found clot reaction time (R) in minutes as well as time to maximum rate of thrombus generation (TMRTG) in minutes to be significantly decreased in female migraine-with-aura at baseline compared to healthy age-matched controls (*P* < 0.05). Results have been summarised in Supplementary Table 1 and Figure 2.

### Erythrocyte Morphology

Axial ratios (major axis: minor axis, arbitrarily determined) are used to compare red blood cell shapes while circumferences are used to compare red blood cell sizes. Examples of Cellanyzer v 1.1 axial ratio and circumference determination are shown in **Figure 4**.

**Figure 3:**
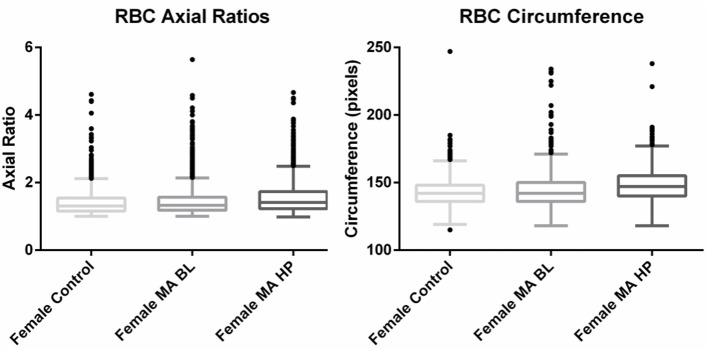
Tukey box plots of the red blood cell (RBC) axial ratios and circumferences of female migraine with aura patients compared to age-matched controls.

**Figure 4:**
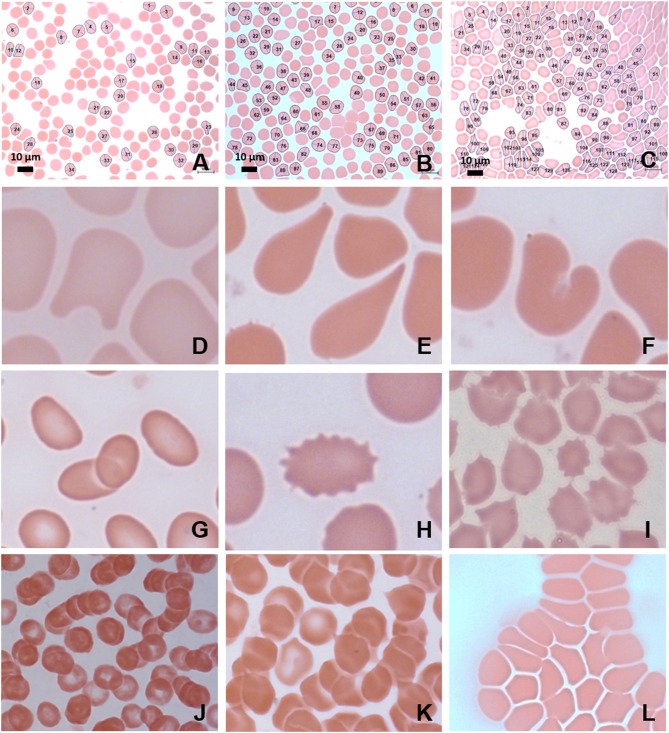
MB/E blood smears of red blood cells with major axis, minor axis, and circumference determined by Cellanyzer software. **(A)** Female control, **(B)** female migraine aura baseline, **(C)** female migraine aura headache phase. Aberrant erythrocyte morphologies widespread in the migraine population. **(D)** Helmet cell; **(E)** teardrop cell; **(F)** irregularly contracted cell; **(G)** elliptocytes; **(H)** echinocyte; **(I)** acanthocytes (spur cells); **(J)** microrouleaux formation; **(K)** microrouleaux with surface projections; **(L)** RBC Agglutination.

Circumferences of erythrocytes were measured as number of pixels (**Figure 4**). Analysis of circumferences by Kruskal Wallis with Dunn's post-test were found to be increased during the headache phase (*P* < 0.0001) of migraine (95% CI [148.00, 149.10]) compared to healthy age-matched controls (95% CI [142.30, 143.40]). There were no significant differences in circumference between headache phase and baseline. A linear model with a random intercept for each of the patients in the female control group, the FMA baseline group as well as the FMA headache phase group (24 subjects), with robust standard errors was used to model circumference in order to consider for between-patient variation. The means of circumference for each of the three groups were estimated from the model. Tests were conducted to test the equality of the migraine group effects after the models were run, which found RBC circumferences to be significantly greater in the headache phase compared to control (*P* = 0.013) as well as compared to baseline (*P* = 0.0290) (95% CI [140.16, 154.87]). Results of Cellanyzer v 1.1 analysis are summarised in Table 1 and Figure 3.

**Table 1:** Summary of results of light microscopy for red blood cell axial ratios and circumferences.

**RBC axial ratios: D'Agostino and pearson:** ***P*** **<** **0.0001**					
**RBC AXIAL RATIOS: MANN-WHITNEY TEST**					
**Group**			***P*****-value**		
Female control vs. FMA BL			0.0107		
Female control vs. FMA HP			**<0.0001**		
**WILCOXON MATCHED-PAIRS SIGNED RANK TEST**					
**Baseline group**	**Headache phase group**		***P*****-value**	**Correlation coefficient**	
FMA BL	FMA HP		**>0.9999**	Spearman	−0.1141
**QUINTILE REGRESSION MODEL FOR CONDITIONAL MEDIAN OF AXIAL RATIOS**					
**Group**		**Beta (95% CI)**		***P*****-value**	
FMA baseline		1.33 (1.31, 1.34)		0.2620	
FMA headache phase		1.42 (1.39, 1.44)		<0.0001	
**Intercept**		1.31 (1.29, 1.33)		<0.0001	
**RBC circumference: D'Agostino and pearson:** ***P*** **< 0.0001**					
**RBC CIRCUMFERENCE: KRUSKAL-WALLIS ANOVA**					
**Group**				***P*****-value**	
Female control vs. FMA BL vs. FMA HP				**<0.0001**	
**Median (25%; 75%)**	**Control group**		**Migraine group**		***P*****-value**
**BASELINE: DUNN'S POST-TEST**					
	Female control	142 (136; 148)	FMA BL	142 (136; 150)	**0.4223**
**HEADACHE PHASE: DUNN'S POST-TEST**					
	Female control	142 (136; 148)	FMA HP	147 (140; 155)	**<0.0001**
**WILCOXON MATCHED-PAIRS SIGNED RANK TEST**					
**Baseline group**	**Headache phase group**		***P*****-value**	**Correlation coefficient**	
FMA BL	FMA HP		**>0.9999**	Spearman	0.0587
**LINEAR MIXED MODEL WITH RANDOM INTERCEPTS FOR CIRCUMFERENCES**					
**Group**		**Beta (95% CI)**		***P*****-value**	
FMA baseline		143.51 (141.04, 145.99)		0.545	
FMA headache phase		147.52 (144.91, 150.11)		0.0113	
**Intercept**		142.26 (139.04, 145.48)		**<0.0001**	

*The significant values to be in bold*.

It was found that the axial ratios of red blood cells of the female migraineurs were increased during the headache phase (95% CI [1.53, 1.58]) of migraine compared to healthy age-matched controls (95% CI [1.39, 1.44]) (*P* < 0.0001); with no significant change in axial ratios from baseline to the headache phase A quantile regression model, modelling the median, with robust standard errors was used to model axial ratio in order to account for non-normality, outliers and for the fact that not all observations are independent. Tests were conducted to test the equality of the migraine group effects after the models were run, which found axial ratios to be significantly greater during the headache phase compared to control (*P* < 0.0001) as well as compared to baseline (*P* < 0.0001) (95% CI [1.36, 1.47]).

This would indicate that the red blood cells of migraineurs show deviation from the normal discoid shape producing cells that are elongated along the major axis around the time of a headache attack.

Having used light microscopy to establish that the red blood cells of migraineurs were of a significantly different size and shape from those of healthy individuals, we studied this further using both light microscopy and the more sensitive method of scanning electron microscopy.

Red blood cells were considered in terms of shape, size, colour, inclusions, and arrangement. Analysis under light microscopy (Zeiss AXIO Imager M2, 100 X) and SEM (Zeiss Cryo CR-Beam Ultra-resolution SEM) showed widespread poikilocytosis throughout the FMA cohort at baseline and persisting into the headache phase compared to age-matched healthy individuals. Poikilocytosis is the presence of abnormally shaped red blood cells which often occurs with anisocytosis—the presence of unequally sized red blood cells. The various alterations of red blood cell morphology seen under light microscopy are illustrated in Figure 4.

**Figure 5:**
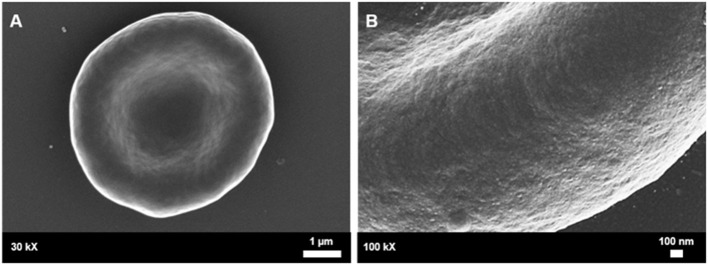
Healthy control red blood cell morphology. **(A)** A healthy red blood cell is discoid in shape with a large biconcave central indentation. **(B)** The external surface of the membrane has an undulating quality.

**Figure 6:**
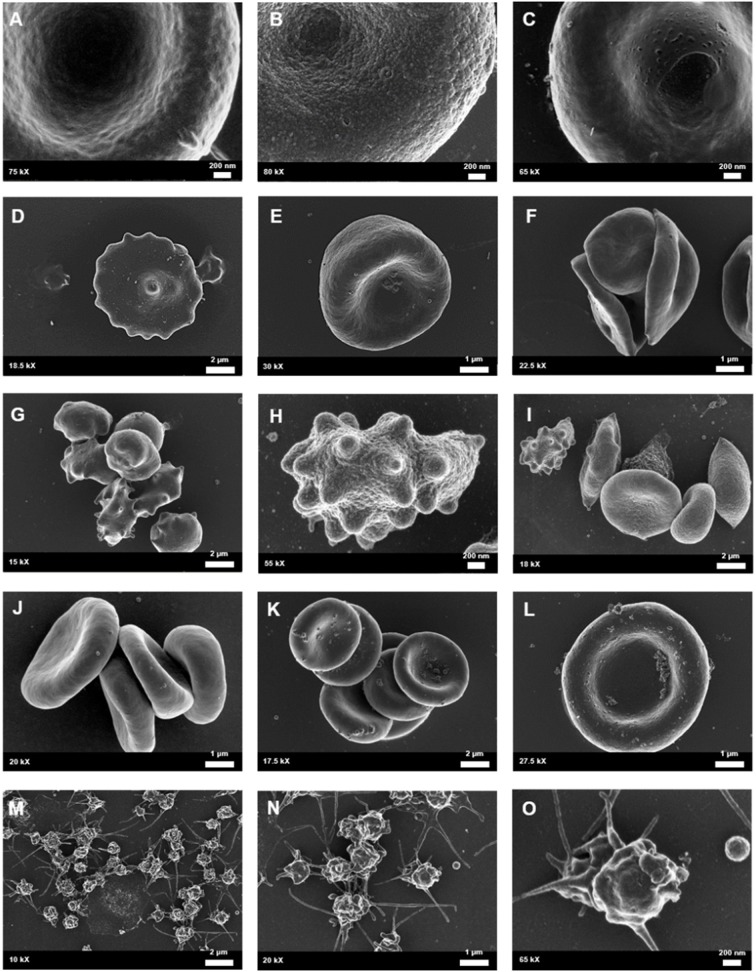
Whole blood of female migraineurs with aura through the phases of migraine. RBC membrane surface ranged from the normal undulating quality **(A)** to granular in texture **(B)** and membranes with pitting defects **(C)**. The presence of pathological erythrocyte morphologies was widespread and included leptocytes **(D)**, stomatocytes **(E)**, and elliptocytes **(F)**. The greatest marker of pathology was the increased presence of RBCs in various stages of eryptosis **(G–I)** compared to healthy age-matched controls. Increased RBC aggregability was also seen **(F,J,K)** as well as protein aggregation on RBC surfaces **(J–L)**. Platelets were also found to be innately activated in whole blood samples of migraineurs, with many forming dense aggregations and platelet thrombi **(M–O)**.

Scanning electron microscopy analysis of whole blood was used to further analyse red blood cell morphologies in samples drawn from the female migraine sufferers at baseline and during headache phase. The various alterations from normal morphology (Figure 5) are shown in Figure 6. The membranes of migraineur erythrocytes ranged from the normal, healthy undulating quality to membranes that were granular in texture or were marked by pitting defects. Increased red blood cell aggregability as well as protein adhesion on red blood cells indicated changes in the electrical potentials across these red blood cell membranes. The red blood cells of females affected by migraine with aura were furthermore widely characterised by the loss of the normal discoid shape. Of special interest was the much increased presence of erythrocytes in various stages of eryptosis (17)—programmed suicide of erythrocytes that can be signalled for by excessive oxidative stress because of persistent systemic inflammation.

Analysis under SEM indicated that the platelets of migraineurs were innately activated with several forming dense aggregations reminiscent of the beginning of a platelet clot. In several native whole blood migraineur samples (i.e., without addition of clot-inducing reagents, such as thrombin) fibrin fibres were found. The combined evidence of extraordinary platelet activation and innate fibrin polymerisation indicate innate activation of the coagulation cascade in episodic migraine.

### Fibrin Morphology and Amyloidogenesis

**Figure 7:**
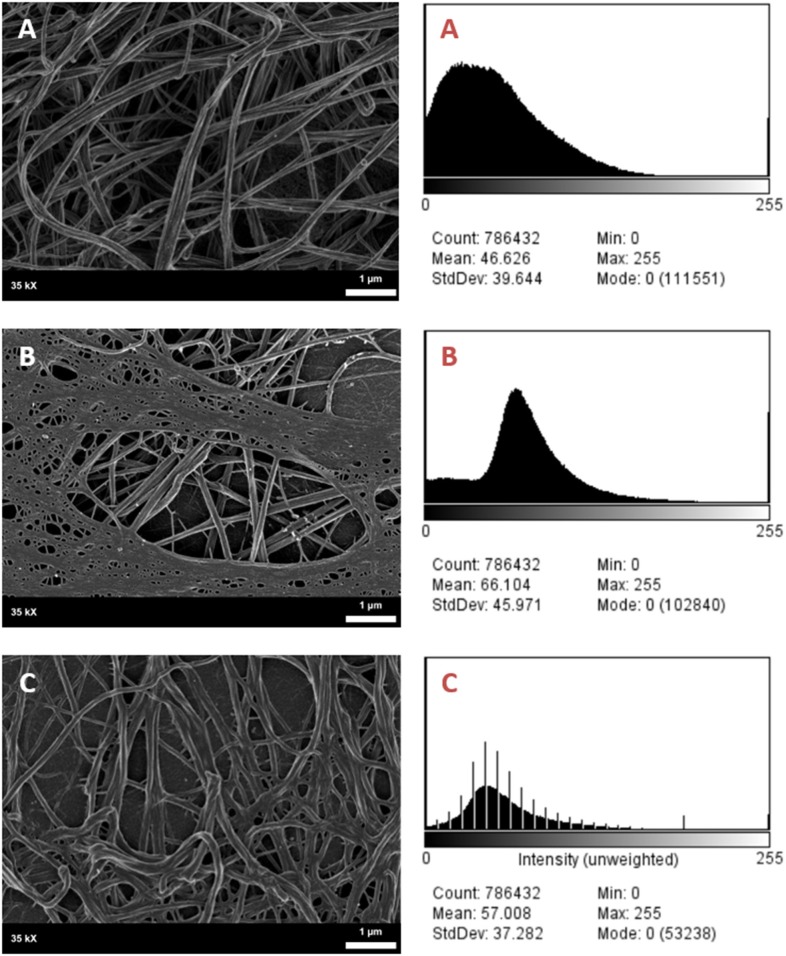
SEM Micrographs of female fibrin clots and corresponding histograms of 8-bit pixel intensity. **(A)** Healthy female control, **(B)** female migraineur with aura at baseline, **(C)** same female migraineur during headache phase.

It was found that the fibrin network micrographs of female migraineurs at baseline (95% CI [0.77, 0.84]) have slightly decreased coefficients of variation compared to those of healthy age-matched controls (95% CI [0.80, 0.84]), with significantly decreased coefficients of variation during the headache phase (95% CI [0.72, 80]) (*P* < 0.0001). Decreased variation indicates fewer black pixels (absence of fibrin) compared to grey pixels (fibrin) and thus denser fibrin networks. Furthermore, it was found that there was significant difference in the density of fibrin networks formed from baseline PPP and headache phase PPP (Table 2). Example micrographs with corresponding histograms are shown in Figure 7.

**Table 2:** Summary of results of 8-bit pixel intensity coefficient of variation analysis of SEM micrographs and of confocal micrographs of fibrin clots treated with the amyloid marker ThT.

**SEM analysis of fibrin network density**						
**Mean ± SEM**	**Control group**		**Migraine group**		***P*-value**	**Significant**
**BASELINE**						
	Female control	0.8207 ± 0.0106	Female migraine aura	0.8059 ± 0.0168	0.4885	No
**HEADACHE PHASE**						
	Female control	0.8207 ± 0.0106	Female migraine aura	0.7577 ± 0.0196	**0.01**	**Yes**
**Baseline group**	**Headache phase group**		***P*****-value**	**Significant**	**Correlation coefficient**	
**PAIRED ANALYSIS**						
Female migraine aura	Female migraine aura		**0.0331**	**Yes**	*r*	0.2698
**Confocal analysis of β-amyloid composition of fibrin networks**						
**Median (25%; 75%)**	**Control group**		**Migraine group**		***P*****-value**	**Significant**
**BASELINE**						
	Female control	1.03 (0.95; 1.19)	Female migraine aura	0.80 (0.69; 1.00)	**0.0002**	**Yes**
**HEADACHE PHASE**						
	Female control	1.03 (0.95; 1.19)	Female migraine aura	1.00 (0.84; 1.32)	0.52	No
**Baseline group**	**Headache phase group**		***P*****-value**	**Significant**	**Correlation coefficient**	
**PAIRED ANALYSIS**						
Female migraine aura	Female migraine aura		**0.0006**	**Yes**	Spearman rs	0.1407

*The significant values to be in bold*.

**Figure 8:**
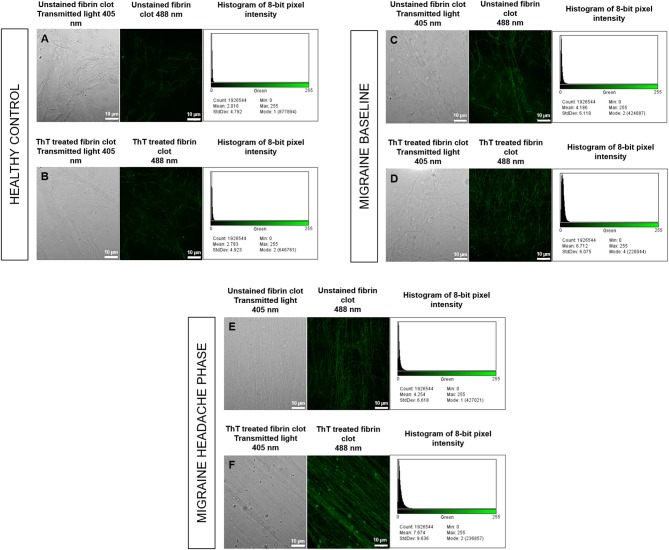
Unstained fibrin clots in comparison to ThT treated fibrin clots with histograms of 8-bit pixel intensity for a healthy control **(A,B)**, female migraine-with-aura at baseline **(C,D)** and female migraine-with-aura during the headache phase **(E,F)**. Beta-amyloid visibility was far improved by addition of ThT to PPP, as seen in **(D,F)**.

Confocal micrographs of ThT-treated fibrin clots taken at 63 X were analysed for 8-bit pixel intensity using the histogram function of FIJI. Example micrographs with corresponding histograms are shown in Figure 8.

Confocal microscopy was first used to view fibrin clots under transmitted light (405 nm) before fluorescence was observed at an excitation wavelength of 488 nm. It has been found that amyloid proteins autofluorescence (31), and this was assessed in order to detect any foci of fibrin autofluorescence (32), and also to determine whether these foci corresponded with fluorescence from ThT binding to areas of β-amyloid misfolding. Some images are shown in Figure 8 and indicate that auto-fluorescent foci are indeed areas of β-amyloid misfolding.

Analysis showed that the fibrin clots of female migraineurs with aura had significantly higher numbers of amyloid fibrils rich in β-sheets at baseline (95% CI [0.80, 0.99]) than did healthy age- and gender-matched controls (95% CI [0.99, 1.16]) (*P* < 0.001). This did not persist into the headache phase (95% CI [1.00, 1.21]) and the presence of amyloid fibrils rich in β-sheets was found to be significantly different between baseline and headache phase. Results are summarised in Table 2.

## Discussion

The results of TEG® analysis of the viscoelastic profiles of the female migraineurs through the phases of migraine compared to healthy age-matched controls indicate that migraine is accompanied by a hypercoagulable state with pronounced hypercoagulability during the headache phase. These results echo the work of Nielsen (33) and Tietjen and Collins (5).

Tietjen and Collins (5) and Tietjen (7, 12) described a model of migraine pathogenesis whereby migraine gives rise to endothelial dysfunction in the arteries closely associated with the trigeminal nerve, promoting a hypercoagulable inflammatory state which induces further endothelial dysfunction and recurring episodic or chronic migraine (5, 7, 12). This was supported by Nielsen et al. (33) who reported that chronic migraineurs showed significantly decreased clotting initiation times, increased velocity of clot formation and greatly increased clot strength, all of which indicate a hypercoagulable state in chronic migraine. Virchow's Triad has been used in medicine and pathology since the 1900's to define the cause and risk for thromboembolism within three parameters, namely endothelial dysfunction, hypercoagulability, and stasis (34, 35). Stasis, such as may be experienced during immobility or under anaesthesia, is considered a transient risk factor (34). As such, evaluation of thromboembolic risk in migraine patients relies on evaluating endothelial function and coagulability (35).

The significant differences in the whole blood of headache phase indicated that clot strength, clot elasticity and the shear elastic modulus strength of clots are greater during the headache phase of migraine with aura, with decreased clotting times. In simple terms, patients experiencing a migraine headache are at risk of forming rapidly growing, large, strong clots that are more resistant to being deformed or broken down.

Changes in red blood cell morphology can manifest as variation in size (microcytic, normocytic, macrocytic), variation in distribution (rouleaux formation and agglutination) and shape variation (poikilocytosis) (16). Lippi et al. (6) suggested a model whereby erythrocytosis promotes hyperviscosity, hypoxia, and vasoconstriction in migraine. Based on their model it is plausible that the changes to erythrocyte morphology which we observed here may contribute to the hypercoagulability we have noted in the TEG results. Analysis of axial ratios and circumferences of female migraineur erythrocytes quantitatively proved macrocytosis and shape variation in episodic migraine. Shape variation was further qualitatively proven through morphological analysis. Our results echo those of Celikbilek et al. (36), who reported higher red cell distribution width in migraine.

Poikilocytosis, in its various forms, can be a sign of a variety of conditions including advanced liver disease, hyposplenism, pyruvate kinase deficiency, anaemia of various origins, oxidative haemolysis, renal failure, and haemoglobinaemias (37, 38). This is a result of the sensitivity of red blood cells to pathology, a characteristic which makes them useful as health indicators (16). A diagnosis of a specific condition would require a predominance of a form of poikilocytosis. The presence of polymorphism in the migraine cohort makes it more likely that a chronic inflammatory state exists in migraine causing biochemical and biophysical disruptions to erythrocyte metabolism, function and morphology (16). Pretorius et al. (16) define biochemical changes as those affecting molecular arrangement and membrane ultrastructure, while biophysical changes affect shape and elasticity or rigidity. Biochemical changes lead to biophysical changes (16). It is thus the biophysical properties of red blood cells that determine deformability and rheology. The changes seen under light microscopy in the red blood cell morphologies of migraine patients mostly indicated biophysical changes, however, agglutination and microrouleaux formation (i.e., variations in distribution) indicate aberration at the biochemical level.

One of the protein classes maintained in the erythrocyte membrane by cholesterol are the sialylated glycoproteins. These glycoproteins are responsible for the negative electric potential across the cell membrane, and thus the repulsive forces between red blood cells that allow for passage of individual cells through the microvasculature (18, 39). If pathological changes affect the sialylated glycoproteins the negative membrane potential may be lost and erythrocytes become attracted to each other, and in the case of rouleauxs, become “stacked” (40). This and other biochemical changes which affect the cell membrane result in altered flexibility and decreased movement of cells through the microvasculature (16, 39). The major causes of widespread variation in size, shape and distribution are oxidative stress (which is in line with the model suggested by Lippi et al.) and upregulation of inflammatory molecules following hyperosmotic shock; both processes accompany chronic systemic inflammation and lead to eryptosis (6, 13, 16–19).

Eryptosis is the programmed suicidal cell death of erythrocytes, occurring in the absence of mitochondria or nuclei. Eryptosis is predominantly triggered by exposure to reactive oxygen species and hyperosmotic shock. Hyperosmotic shock activates the cyclooxygenase (COX) enzyme system as well as phospholipase A2 (PLA2). COX activation leads to production of prostaglandin E2 (PGE2), which in turn causes the opening of calcium permeable non-selective cation channels. PLA2 activation leads to release of platelet activating factor (PAF) and later ceramide production. Ceramide increases calcium sensitivity of the cell (15, 17, 41).

Oxidative stress also increases calcium entry through cation channels (via PGE2 action). The net calcium influx following oxidative stress and hyperosmotic shock leads to the activation of calpain; the translocation of phosphatidylserine to the outer membrane leaflet, i.e., the “PS flip”; and the activation of potassium and chloride channels. The PS flip in combination with the activation of calcium permeable non-selective cation channels via PGE2 leads to the first stage of eryptosis, cell membrane scrambling. Calpain is an endopeptidase that degrades membrane proteins, resulting in the second stage of eryptosis, membrane blebbing. Lastly, net efflux of potassium and chloride causes membrane hyperpolarisation and the net loss of water, leading to the last stage of eryptosis, cell shrinkage (15, 17, 41).

In contrast to those of controls, cells in all three stages of eryptosis—membrane scrambling, cell shrinkage and membrane blebbing—were widespread both at baseline and during the headache phase of migraine as evidenced using scanning electron microscopy. The cumulative evidence of the analysis of whole blood cells supports the theory of migraine as a chronic systemic inflammatory condition, which both results from and will produce high levels of oxidative stress (16). Several factors affect coagulability of both healthy individuals and those suffering from disease conditions. Advancing age, female gender, combined oral contraceptive use, and low-normal haematocrit are known to be procoagulant (42).

Investigation by scanning electron microscopy furthermore indicated increased platelet activation in this cohort, although platelet function was not a main endpoint for our study. Borgdorff and Tangelder (43) proposed that increased platelet activation, reactivity and aggregability in the headache phase of migraine plays a vital role not only in thromboembolic risk in migraine but also in the degree of vasoconstriction owing to serotonin release from platelets (43). It is also known that platelet activation during blood coagulation results in the release of the glycoprotein thrombospondin which co-polymerises with fibrin, possibly modulating clot structure (23). With platelets affecting both rheology and coagulability it may of great use to further investigate the role of platelet activation in migraine-with-aura at a later time

From the perspective of Virchow's Triad the results of viscoelastic analysis would suggest significant risk of thromboembolism in FMA patients during the headache phase of migraine, as is supported by the evidence of biochemical and biophysical changes to erythrocytes. Future enquiry would need to determine how shear modulus and elasticity could be inferred to seek to quantitate the risk for embolisation. It has recently been shown that microembolisation may be the cause of cortical spreading depression experienced in migraine with aura, and may be the causative link between migraine and a variety of cerebrovascular disorders (4). The hypercoagulable viscoelastic profiles of migraineurs, especially those measured during the headache phase, support the possibility of microembolisation as per Virchow's triad.

Following changes in primary structure (such as seen with fibrinogen oxidation) or under great mechanical stress the tertiary structure of fibrinogen can be altered from mainly α-helices to mainly β-sheets. This same alteration in architecture has been seen in other proteins undergoing amyloidogenesis (44). Amyloidogenesis is the process whereby protein misfolding gives rise to insoluble proteinaceous fibrils. This process has been implicated in various conditions, such as Alzheimer's, prion diseases, and the systemic amyloidosis (28). It has been demonstrated that the β-Amyloid formed in Alzheimer's disease associates with the β-chain C termini fibrinogen inducing oligomerisation and resulting in the formation of structurally abnormal fibrin fibrils with the resultant fibrin clots being resistant to fibrinolysis (45, 46). Recent work by Pretorius et al. has indicated that the addition of substoichiometric levels of Gram-negative bacterial lipopolysaccharide (LPS), lipoteichoic acid (LTA) from Gram-Positive bacteria as well as molecules like iron, may in part, induce amyloidogenesis during blood clotting. Ultrastructural analysis implied that in this amyloidogenic state fibrin was in an autocatalysed β-sheet-rich form that resembled what had been seen in plasma from individuals with inflammatory and amyloid diseases, indicating LPS, as one of the possible reasons for misfolding of fibrinogen that any point the possibility that LPS might have an etiological involvement (44, 47, 48). However, a systems biology approach must be taken when unraveling the protein misfolding process, as the involvement of bacterial inflammagens and other dysregulated inflammatory molecules in disease etiology is complex and rarely the result of a single dysregulated molecule or event.

The oxidative damage seen in this cohort reflects the pattern of ozone oxidation described by Rosenfeld et al. (21). According to this model, oxidisation results in the formation of reactive functional groups and conformational changes of the D regions of fibrinogen. This in turn exposes new reaction sites allowing for auto-catalysation with inhibition of longitudinal polymerisation. Fibrin thus polymerises in a tail-to-tail pattern as opposed to the normal middle-to-tail pattern (21, 49). In this cohort, these changes may be attributable to β-amyloidogenic misfolding of the tertiary structure of fibrin as shown by ThT binding under confocal microscopy.

**Figure 9:**
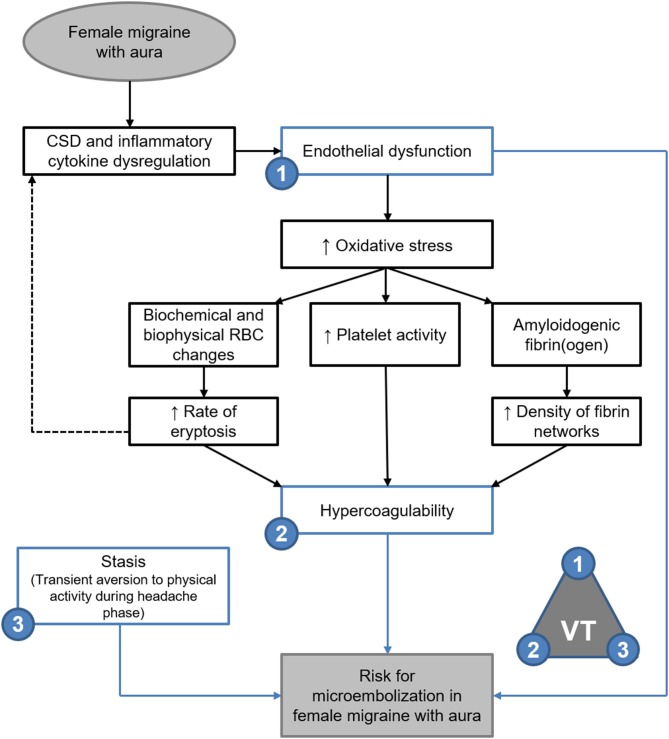
Endothelial dysfunction, hypercoagulability and stasis in FMA support the theory of microembolisation described by Dalkara et al. (4) as per Virchow's triad (VT). Evidence of amyloidogenic blood clotting would further indicate that such microemboli are resistant to degradation.

Elasticity and stiffness of fibrin networks are conferred through factor XIII-catalysed α-α crosslinking between individual fibres, which promotes lateral aggregation and formation of branching points (21, 49–51). As such it would be expected that fibrin networks rich in β-amyloidogenic misfolded protein fibrils would exhibit decreased elasticity with decreased lateral aggregations and branching points—as described in the oxidation model described by Weigandt et al. (22) However, it has been established that the blood clots formed in amyloidogenic states are resistant to degradation by tissue plasminogen activator (tPA) (45, 52). reflecting the insoluble nature of the proteinaceous fibrils (28, 45, 46). Thus, the findings presented here support the microembolisation theory of migraine described by Dalkara et al. (4), and are summarised in Figure 9.

Following previous work into the causative role of bacterial inflammagens in amyloidogenesis it may be valuable to investigate the possibility of a link between migraine and conditions of gut dysbiosis, which are characterised by elevated serum levels of bacterial LPS and other such related molecules (53–55).

Further advancement of this study would be achieved by increasing the sample size, using the TEG data as a guide at least 48 participants would be required to have a 90% power to detect a 15% difference between the female episodic migraine-with-aura and control groups. Stage of ovarian and menstrual cycles, as well as contraceptive method, could also be controlled for in a larger cohort. Furthermore, we would aim to improve the quality of a larger study by evaluating confounding variables, such as obstetric history, body mass index, serum lipid profiles and hypertension.

## Conclusion

In this pilot study, morphological analysis of the whole blood of female migraineurs with aura is suggestive of a systemic inflammatory state both at baseline and during the headache phase characterised by macrocytosis, poikilocytosis and eryptosis. The observed variations in erythrocyte size, shape and distribution are most likely a result of oxidative stress. This observed inflammatory pathology is the likely cause of the known hypercoagulability in FMA, which was most prominently observed in this cohort during the headache phase. This hypercoagulable state may be a result of aberrant fibrin(ogen) polymerisation kinetics; which if present in a larger cohort would support theories of micro-embolisation in migraine with aura. Another trend indicated by the viscoelastic analysis of episodic female migraine-with-aura is that hypercoagulability is more pronounced in platelet poor plasma than in whole blood. This would suggest an inherent change in both fibrinogen and fibrin in these migraineurs, possibly as a result of the oxidative stress evidenced in the red blood cells of these patients. It is known that oxidative damage to fibrinogen can result in a protein misfolding with changes to its tertiary structure visible with confocal microscopy as both auto-fluorescent and amyloid areas, and in SEM as denser and matted fibrin areas. This suggests that in women with episodic migraine-with-aura, abnormal blood clotting takes place. Hopefully, this knowledge may lead to novel diagnostics and therapeutics.

## Data Availability Statement

All datasets generated for this study are included in the article/Supplementary Material.

## Ethics Statement

This study was approved by the Faculty of Health Sciences Research Ethics Committee of the University of Pretoria (protocol number 101/2015) and governed by the Declaration of Helsinki. Written informed consent was obtained from all participants in the form of a participant consent waiver approved by the Faculty of Health Sciences Research Ethics Committee and co-signed by the principal researcher (SV). All participants were over the age of 18 and legally competent. All participants were allocated a unique identifier to ensure anonymity of all samples, microscopy slides, and data held by the University of Pretoria.

## Author Contributions

SV: wrote the paper. JB: technical support. EP: study leader, edited paper, and co-corresponding author. DK: edited the paper and co-corresponding author. All authors reviewed the manuscript.

### Conflict of Interest

The authors declare that the research was conducted in the absence of any commercial or financial relationships that could be construed as a potential conflict of interest.
