# Supplementary material for: A Possible Role of Amyloidogenic Blood Clotting in the Evolving Haemodynamics of Female Migraine-With-Aura: Results From a Pilot Study
Source: Front Neurol. 2019 Nov 26;10:1262. doi: 10.3389/fneur.2019.01262 (PMC6887655; doi:10.3389/fneur.2019.01262)
Supplement: Supplementary file 1 [file Data_Sheet_1.PDF]

Supplementary Material

Supplementary Table 1. Summary of results for the comparison of the viscoelastic profiles of female episodic migraine with aura sufferers to those of healthy age-matched controls

Key: FC = female control, WB = whole blood; PPP = platelet poor plasma, BL = baseline, HP = headache phase, FMA = female migraine-with-aura

R (min)					
Median (25%;75%)	Control Group		Migraine Group		P value
	FC WB	7.50 (6.53;9.75)	FMA BL WB	7.20 (5.80;8.20)	0.47
	FC WB	7.50 (6.53;9.75)	FMA HP WB	6.80 (5.30;7.20)	0.32
	FC PPP	10.95 (8.70;11.55)	FMA BL PPP	6.40 (6.00;8.70)	**0.03**
	FC PPP	10.95 (8.70;11.55)	FMA HP PPP	8.00 (5.20;10.80)	0.16
**PAIRED ANALYSES: R (min)**					
**Baseline Group**	**Headache Phase Group**	**P value**	**Correlation Coefficient**		
FMA BL WB	FMA HP WB	0.28	Spearman rs		0.12
FMA BL PPP	FMA HP PPP	0.64	Spearman rs		-0.14
**K (min)**					
Median (25%;75%)	**Control Group**		**Migraine Group**		**P value**
	FC WB	3.55 (2.85;4.25)	FMA BL WB	2.80 (2.30;3.20)	0.1143
	FC WB	3.55 (2.85;4.25)	FMA HP WB	2.40 (1.90;3.40)	0.0945
	FC PPP	3.80 (2.35;4.28)	FMA BL PPP	2.00 (1.10;3.20)	0.1099
	FC PPP	3.80 (2.35;4.28)	FMA HP PPP	2.20 (1.20;3.20)	0.1040
**PAIRED ANALYSES: K (min)**					
**Baseline Group**	**Headache Phase Group**	**P value**	**Correlation Coefficient**		
FMA BL WB	FMA HP WB	0.81	Spearman rs		-0.01
FMA BL PPP	FMA HP PPP	0.76	Spearman rs		-0.06
**α (degrees)**					
Median (25%;75%)	**Control Group**		**Migraine Group**		**P value**

	FC WB	50.65 (41.25;59.83)	FMA BL WB	52.8 (48.5;70.3)	0.25
	FC WB	50.65 (41.25;59.83)	FMA HP WB	62.5 (56.9;67.7)	0.05
	FC PPP	63.45 (55.03;67.68)	FMA BL PPP	67.5 (59.1;75.9)	0.36
	FC PPP	63.45 (55.03;67.68)	FMA HP PPP	66.7 (59.0;75.6)	0.37
**PAIRED ANALYSES: α (degrees)**					
**Baseline Group**	**Headache Phase Group**		**P value**	**Correlation Coefficient**	
FMA BL WB	FMA HP WB		0.3750	Spearman rs	0.60
FMA BL PPP	FMA HP PPP		0.8311	Spearman rs	0.33
**MA (mm)**					
Median (25%;75%)	**Control Group**		**Migraine Group**		**P value**
	FC WB	45.25 (43.20;48.80)	FMA BL WB	53.4 (45.8;60.4)	0.12
	FC WB	45.25 (43.20;48.80)	FMA HP WB	56.0 (43.5;63.2)	0.21
	FC PPP	23.90 (21.85;32.33)	FMA BL PPP	29.2 (26.2;41.3)	0.17
	FC PPP	23.90 (21.85;32.33)	FMA HP PPP	30.9 (23.9;39.7)	0.36
**PAIRED ANALYSES: MA (mm)**					
**Baseline Group**	**Headache Phase Group**		**P value**	**Correlation Coefficient**	
FMA BL WB	FMA HP WB		0.7188	Spearman rs	0.49
FMA BL PPP	FMA HP PPP		0.8311	Spearman rs	0.33
**G (dynes/cm2)**					
Median (25%;75%)	**Control Group**		**Migraine Group**		**P value**
	FC WB	4150 (3825;4775)	FMA BL WB	5700 (4200;7600)	0.11
	FC WB	4150 (3825;4775)	FMA HP WB	6400 (3800;8600)	0.21
	FC PPP	1550 (1400;2375)	FMA BL PPP	2100 (1800;3500)	0.10
	FC PPP	1550 (1400;2375)	FMA HP PPP	2200 (1600;3300)	0.15
**PAIRED ANALYSES: G (dynes/cm2)**					

Baseline Group	Headache Phase Group		P value	Correlation Coefficient	
FMA BL WB	FMA HP WB		0.65	Spearman rs	0.50
FMA BL PPP	FMA HP PPP		0.68	Spearman rs	0.36
**E (dynes/cm2)**					
	**Control Group**		**Migraine Group**		**P value**
Median (25%;75%)	FC WB	82.80 (76.05;95.48)	FMA BL WB	114.8 (84.5;152.4)	0.10
	FC WB	82.80 (76.05;95.48)	FMA HP WB	127.3 (76.9;171.9)	0.22
	FC PPP	31.45 (27.95;48.15)	FMA BL PPP	41.2 (35.5;70.4)	0.07
	FC PPP	31.45 (27.95;48.15)	FMA HP PPP	44.7 (31.4;65.9)	0.16
**PAIRED ANALYSES: E (dynes/cm2)**					
**Baseline Group**	**Headache Phase Group**		**P value**	**Correlation Coefficient**	
FMA BL WB	FMA HP WB		0.70	Spearman rs	0.49
FMA BL PPP	FMA HP PPP		0.70	Spearman rs	0.33
**MRTG [(dynes/cm2/s)/2]**					
	**Control Group**		**Migraine Group**		**P value**
Median (25%;75%)	FC WB	3.49 (2.70;4.69)	FMA BL WB	5.54 (3.57;6.22)	0.10
	FC WB	3.49 (2.70;4.69)	FMA HP WB	4.68 (4.39;6.27)	0.07
	FC PPP	4.40 (3.62;6.53)	FMA BL PPP	9.30 (5.25;12.76)	0.13
	FC PPP	4.40 (3.62;6.53)	FMA HP PPP	6.53 (3.97;10.27)	0.25
**PAIRED ANALYSES: MRTG [(dynes/cm2/s)/2]**					
**Baseline Group**	**Headache Phase Group**		**P value**	**Correlation Coefficient**	
FMA BL WB	FMA HP WB		> 0.999	Spearman rs	0.26
FMA BL PPP	FMA HP PPP		0.4648	Spearman rs	0.02
**TMRTG (min)**					
	**Control Group**		**Migraine Group**		**P value**
Median (25%;75%)	FC WB	8.96 (8.31;12.11)	FMA BL WB	9.33 (6.92;11.75)	0.66
	FC WB	8.96 (8.31;12.11)	FMA HP WB	9.33 (7.75;10.33)	0.73

	FC PPP	12.09 (10.54;13.06)	FMA BL PPP	8.92 (6.83;10.33)	**0.04**
	FC PPP	12.09 (10.54;13.06)	FMA HP PPP	9.17 (7.08;11.25)	0.07
**PAIRED ANALYSES: TMRTG (min)**					
**Baseline Group**	**Headache Phase Group**	**P value**	**Correlation Coefficient**		
FMA BL WB	FMA HP WB	0.78	Spearman rs		0.73
FMA BL PPP	FMA HP PPP	0.58	Spearman rs		-0.10
**TTG (dynes/cm2)**					
	**Control Group**		**Migraine Group**		**P value**
Median (25%;75%)	FC WB	413.1 (380.3;477.2)	FMA BL WB	575.8 (422.5;678.8)	0.0967
	FC WB	413.1 (380.3;477.2)	FMA HP WB	647.5 (386.1;842.7)	0.1626
	FC PPP	159.5 (140.0;241.8)	FMA BL PPP	201.2 (177.9;355.3)	0.1275
	FC PPP	159.5 (140.0;241.8)	FMA HP PPP	223.2 (157.7;329.5)	0.1626
**PAIRED ANALYSES: TTG (dynes/cm2)**					
**Baseline Group**	**Headache Phase Group**	**P value**	**Correlation Coefficient**		
FMA BL WB	FMA HP WB	0.4648	Spearman rs		0.50
FMA BL PPP	FMA HP PPP	0.8311	Spearman rs		0.29
